# Supplementary material for: Optimizing Procedures for Antioxidant Phenolics Extraction from Skin and Kernel of Peanuts with Contrasting Levels of Drought Tolerance
Source: Foods. 2022 Feb 3;11(3):449. doi: 10.3390/foods11030449 (PMC8834250; doi:10.3390/foods11030449)
Supplement: Supplementary file 1 [file foods-11-00449-s001.zip › foods-1545552-supplementary.pdf]

Table S1. Experimental design applied to kernels and skins of drought tolerant (BR1) and drought sensitive (LViPE-06) peanut genotypes and responses of the dependent variables.

Run	Independent variables				Dependent variables					
	Coded value		Real value		TPC (µg GAE/mg)		ABTS (µmol TE/g)		ORAC (µmol TE/g)	
	Temp. (°C)	EtOH (%)	Temp. (°C)	EtOH (%)	BR1	LViPE-06	BR1	LViPE-06	BR1	LViPE-06
Kernels										
1	-1	1	46	78	1.49	1.23	38.09	39.05	126.52	78.97
2	1	1	74	78	1.66	1.45	42.22	43.65	164.33	110.89
3	-1	-1	46	42	3.77	2.84	57.47	59.18	223.23	121.26
4	1	-1	74	42	4.16	3.51	63.03	64.81	193.48	111.72
5	0	0	60	60	1.91	1.67	53.53	55.43	186.50	110.23
6	0	0	60	60	1.82	1.56	49.86	50.59	171.55	83.86
7	0	0	60	60	1.88	1.49	52.14	53.03	183.46	83.39
8	0	0	60	60	1.88	1.56	53.74	55.32	178.25	91.19
9	0	0	60	60	1.87	1.60	53.35	54.91	180.78	94.86
10	-1.41	0	40	60	1.76	1.46	50.12	51.67	177.82	90.08
11	0	-1.41	60	35	4.27	3.63	64.81	66.64	32.02	106.20
12	1.41	0	80	60	3.75	3.54	58.50	59.89	72.36	131.37
13	0	1.41	60	85	1.25	1.10	26.77	27.50	8.33	42.26
Skins										
1	-1	1	46	85.5	45.39	92.42	748.34	1150.13	620.38	650.53
2	1	1	74	85.5	49.87	91.47	680.63	1039.99	806.00	721.71
3	-1	-1	46	14.5	40.65	84.19	591.97	953.72	624.35	563.30
4	1	-1	74	14.5	37.29	68.28	545.98	742.09	550.06	554.97
5	0	0	60	50	62.50	93.12	850.45	1070.93	803.08	771.90
6	0	0	60	50	67.79	107.33	934.36	1104.29	887.56	771.36
7	0	0	60	50	68.93	101.20	1000.95	1049.62	857.41	741.30
8	0	0	60	50	68.62	108.24	969.58	1148.04	753.46	698.17
9	0	0	60	50	74.93	95.92	973.40	1168.62	816.95	731.29
10	-1.41	0	40	50	70.55	101.61	949.81	1225.25	900.08	758.66
11	0	-1.41	60	0	32.02	61.00	403.88	610.79	406.80	406.87
12	1.41	0	80	50	72.36	96.44	987.40	1043.23	897.67	776.58
13	0	1.41	60	100	8.33	85.63	160.06	977.99	137.54	740.53

GAE: gallic acid equivalent. TPC: total phenolic content. TE: Trolox equivalent.

Table S2. Summary of ANOVA to temperature and % EtOH on the total phenolic content and antioxidant activity of kernels and skins of drought tolerant (BR1) and drought sensitive (LViPE-06) peanut genotypes.

Parameter	R^2		Quadratic sum (QS)		Degrees of freedom (df)		Quadratic mean (QM)		F-test	
	BR1	LViPE-06	BR1	LViPE-06	BR1	LViPE-06	BR1	LViPE-06	BR1	LViPE-06
Total phenolic content										
Kernels	0.94	0.95								
Regression			14.148	10.30196	5	5	2.82967	2.06039	30.5948	24.2889
Lack-of-fit			0.643	0.57788	3	3	0.214217	0.19263	179.637	48.3987
Pure error			0.005	0.01592	4	4	0.001193	0.00398		
Total			14.796	10.89576	12	12				
Skins	0.91	0.91								
Regression			4452.884	2130.312	1	2	4452.884	1065.16	118.784	52.8674
Lack-of-fit			334.231	21.2	7	6	47.74729	3.533	2.44457	0.0784
Pure error			78.128	180.277	4	4	19.532	45.0693		
Total			4865.243	2331.789	12	12				
ABTS										
Kernels	0.97	0.96								
Regression			1243.308	1316.406	3	3	414.436	438.802	84.3817	86.8838
Lack-of-fit			33.794	28.383	5	5	6.7588	5.6766	2.5973	1.3301
Pure error			10.409	17.071	4	4	2.6023	4.26775		
Total			1287.511	1361.860	12	12				
Skins	0.91	0.96								
Regression			772143.2	344821.1	1	3	772143.20	114940.4	124.1863	68.8192
Lack-of-fit			54803.8	4958.3	7	5	7829.114	991.66	2.3044	0.3938
Pure error			13590.0	10073.3	4	4	3397.5	2518.325		
Total			840537.0	359852.7	12	12				
ORAC										
Kernels	0.93	0.85								
Regression			17030.52	4832.976	5	3	3406.104	1610.992	22.39048	12.17188
Lack-of-fit			935.51	982.572	3	5	311.8367	196.5144	9.64319	3.768072
Pure error			129.35	208.610	4	4	32.3375	52.1525		
Total			18095.38	6024.158	12	12				
Skins	0.88	0.90								
Regression			541889.8	131976.6	2	2	270944.9	65988.3	37.91412	38.81779
Lack-of-fit			61004.7	13210.0	6	6	10167.45	2201.667	3.888833	2.323965
Pure error			10458.1	3789.5	4	4	2614.525	947.375		
Total			613352.6	148976.1	12	12				

Table S3. Yield extraction under optimal conditions of kernels and skins of peanut genotypes with varying drought tolerances.

Genotype	Extraction yield (%)
Kernel	
Senegal 55437	15.22±0.26
L7 Bege	11.39±0.72
Senegal 57422	15.12±0.38
L50	13.06±0.49
LViPE-06	16.57±1.32
LGoPE-06	12.89±0.22
FM407B	12.89±0.66
M.407.424B	17.13±0.29
FM.424B	11.48±0.70
Florunner	8.54±0.54
BR1	17.75±2.51
Tatu	14.80±0.65
Porto Alegre	10.73±0.73
BRS151 L7	14.09±0.48
Skin	
Senegal 55437	11.38±0.85
L7 Bege	11.84±0.76
Senegal 57422	14.61±0.67
L50	9.04±0.07
LViPE-06	10.99±1.51
LGoPE-06	10.64±0.42
FM407B	11.09±0.33
M.407.424B	7.97±0.24
FM.424B	11.70±0.44
Florunner	9.23±0.51
BR1	5.48±0.66
Tatu	7.94±0.11
Porto Alegre	13.52±0.33
BRS151 L7	12.86±0.68

Table S4. ANOVA of the total phenolic content (TPC) and reactive oxygen species scavenging activities from optimized extracts of skin and kernel of peanut genotypes with varying levels of drought tolerance.

Source of variation	DF	Mean square											
		Skin						Kernel					
		TPC	$O_2^{\bullet -}$	H_2O_2	HOCl	$ROO^{\bullet}$	$^{\bullet}OH$	TPC	$O_2^{\bullet -}$	H_2O_2	HOCl	$ROO^{\bullet}$	$^{\bullet}OH$
Genotype	13	7581.95**	97.74**	99.69**	0.18**	1541646.92**	0.0009**	17.46**	159.74**	134291.59**	87.17**	62349.33**	3.46**
Error	28	821.25	1.19	3.69	0.01	26532.94	0.00004	0.61	2.04	738.8	3.11	360.76	0.18
Mean		556.81	19.04	35.9	1.95	4326.95	7.54	21.21	15.72	599.96	18.66	295.77	4.9
CV(%)		5.14	5.73	5.35	6.63	3.76	7.91	3.67	9.09	4.53	9.44	6.42	8.74
Bartlett test		0.008**	0.339	0.218	0.068	0.105	0.002**	0.610	0.171	0.316	0.0001**	0.472	0.151

CV: Coefficient of variation; DF: degree of freedom. $p \leq 0.05$.
